# User experience and safety of generative AI-based mental health chatbots: Scoping review protocol

**DOI:** 10.1371/journal.pone.0341631

**Published:** 2026-01-23

**Authors:** Lotenna Olisaeloka, Chris Richardson, Daniel Vigo

**Affiliations:** 1 Department of Psychiatry, Faculty of Medicine, University of British Columbia, Vancouver, Canada; 2 School of Population and Public Health, Faculty of Medicine, University of British Columbia, Vancouver, Canada; Universitas Al Asyariah Mandar, INDONESIA

## Abstract

**Introduction:**

Mental health problems constitute a significant global health challenge due to their rising prevalence and substantial treatment gap. Digital Mental Health Interventions (DMHIs) including mental health chatbots have emerged as promising solutions due to their effectiveness and scalability. Recent advances in Generative Artificial Intelligence (GenAI) have improved the conversational abilities of these chatbots, further amplifying their potential. However, despite instances of inadvertent harm stemming from the unpredictable nature of GenAI, little attention has been paid to user experience and safety of these chatbots.

**Objective:**

This proposed review will explore existing research on GenAI-based mental health chatbots. Specifically, it aims to identify and describe current chatbots, focusing on user experience, safety and risk mitigation strategies.

**Methods:**

The review will follow the Joanna Briggs Institute (JBI) guidelines for conducting scoping reviews. It will also adhere to the Preferred Reporting Items for Systematic Reviews and Meta-Analyses Extension for Scoping Review (PRISMA-ScR). A systematic database search of Medline (PubMed), Scopus, PsycINFO, ACM Digital Library, and IEEE Xplore will be conducted. The database search will be complimented by research-based search engines (Google Scholar and Consensus). Studies focusing on the development, evaluation or implementation of GenAI-based mental health chatbots will be included without limitations to specific disorders or population groups. Two independent reviewers will perform screening and data extraction. The analysis will include descriptive summary and thematic analysis, with results presented in tabular, graphical, and narrative formats.

**Conclusion:**

This review will provide a comprehensive overview of GenAI-based mental health chatbots while identifying innovative practices and knowledge gaps relating to user experience and safety. Findings will inform the ethical development, evaluation and implementation of GenAI-based mental health interventions.

## Background

Mental health and substance use disorders affect over one billion people globally, contributing substantially to disability, premature mortality, and economic burden worldwide [1–3]. Despite effective therapeutic approaches, a significant treatment gap persists with a majority of affected individuals remaining untreated [3,4]. This gap is driven by persistent barriers to care including treatment costs, shortages of trained personnel, geographical inaccessibility, and stigma [3].

Digital Mental Health Interventions (DMHI) have emerged as a promising strategy to address some of these challenges and expand access to care. DMHI encompass a range of technology-based tools such as online platforms, mobile apps, chatbots and virtual reality (VR), designed to deliver mental health services and support [5,6]. Asynchronous and self-guided DMHI, like chatbots are especially promising due to their accessibility and scalability [5,7].

Conversational Agents (CA), commonly called chatbots, are software applications that mimic human conversation through text or voice interactions. These agents have been used to deliver mental health interventions for a variety of conditions including depression, anxiety, eating disorders, and substance use [8–13]. However, user engagement with these tools is often limited, attributable to the lack of personalization and dynamic interaction [14–16].

Personalization—the tailoring of an intervention to a user’s unique context—has been shown to enhance engagement, user experience, and effectiveness [12]. However, traditional mental health chatbots are primarily rule-based, relying on pre-programmed conversational flow which limit their flexibility and ability to personalize responses. While retrieval-based chatbots offer more adaptability, they still depend on pre-scripted responses, which restrict their ability to meet complex user needs [17,18]. In contrast, GenAI mental health chatbots powered by large language models (LLMs) can produce more interactive and contextually relevant responses. This allows for natural and tailored empathetic conversations, which emerging evidence suggest may improve engagement and therapeutic outcomes [19–21].

The emergence of LLMs marked a turning point in the development of sophisticated mental health chatbots capable of human-like support [22,23]. However, the same flexibility and sophistication that enhance personalization and user engagement also introduce novel risks and safety challenges. GenAI chatbots may generate misinformation, produce inappropriate or harmful responses, and exhibit algorithmic bias. Further, their “black box” nature make them unpredictable and less reliable especially in crisis situations [24–26]. These concerns have triggered broader debates about the ethical and safe deployment of GenAI in mental healthcare [27,28].

Despite increasing discourse on the ethical application of GenAI for mental health, there remains limited research on how to design and deploy GenAI-based mental health chatbots in effective and safe ways. Existing reviews in this area largely focus on traditional rule- and retrieval-based models [8,10–12]. Notwithstanding, a recent meta-analysis highlighted the superior efficacy of AI-based mental health chatbots compared to traditional ones, due to their ability to simulate empathetic conversations and personalize interactions [21]. Still, there remains a lack of systematic synthesis examining their characteristics, user experience and safety profiles.

In this review, User Experience (UX) is conceptualized according to the International Organization for Standardization (ISO 9241−210), which defines UX as an individual’s “perceptions and responses resulting from the use and/or anticipated use of a product, system or service.” The ISO notes that UX “includes all the users’ emotions, beliefs, preferences, perceptions, physical and psychological responses, behaviours and accomplishments that occur before, during and after use.” [29]. In the context of DMHIs, this encompasses measures of acceptability, usability, perceived impact, and engagement [9,30]. Emerging research highlights both the appeal and pitfalls of GenAI mental health chatbots: users appreciate the engaging on-demand and non-judgmental support, but also express concerns about unreliable or potentially harmful content, as well as the risk of overdependence [31,32].

User safety is another critical consideration for DMHI and refers to how digital tools minimize harm, uphold data protection and privacy, and promote psychological well-being throughout intervention design and delivery [33]. The World Health Organization in its guidelines for digital interventions calls for safety considerations including assessing benefits and harms, ensuring data privacy, and using evidence to guide implementation [34]. The safety of traditional mental health chatbots previously received limited attention, mainly because the rule- and retrieval-based systems were perceived as low-risk [35]. In contrast, GenAI’s unpredictability poses challenges in ensuring reliable and safe responses which could result in serious consequences [36]. For instance, there have been reports of GenAI chatbots offering harmful advice [37], promoting substance use, and soliciting explicit content from minors [31]. In some cases, persistent interactions with GenAI chatbots have been linked to tragic outcomes, including suicide [38,39]. These incidents underscore the urgent need for robust safety protocols. The American College of Physicians has called for transparency, rigorous testing, and focused research to understand and mitigate AI-related risks in healthcare [40].

This scoping review fills a critical research gap relating to user experience and safety of GenAI-based mental health interventions. While, existing reviews have examined broader applications of AI and large language models (LLMs), none have systematically mapped *Generative AI-based* chatbots or explored how user experience and safety are conceptualized and operationalized within this emerging domain [12,24,41]. The proposed review therefore fills this gap by focusing specifically on LLM-powered chatbots and by integrating both user-centered and safety-oriented perspectives. A preliminary search of MEDLINE, the Cochrane Database of Systematic Reviews, and JBI Evidence Synthesis revealed no published or registered scoping or systematic reviews on this specific topic as of August 2024, when this review was registered.

### Review questions

The proposed review seeks to:

1. Identify and describe Generative AI-based chatbots developed specifically to deliver mental health interventions.
2. Assess how user experience (e.g., acceptability, usability, engagement) are reported in studies of these chatbot interventions.
3. Examine the safety mechanisms and risk mitigation strategies integrated during the development and deployment of these chatbot interventions.

## Methods

The proposed scoping review will follow guidelines outlined in the Joanna Briggs Institute (JBI) manual for scoping reviews [42] and adhere to the Preferred Reporting Items for Systematic Reviews and Meta-Analyses extension for Scoping Reviews (PRISMA-ScR) [43]. The review has been registered in Open Science Framework Registries (doi.org/10.17605/OSF.IO/HSNXA).

### Eligibility criteria

The PCC (population, concept, context) framework recommended by JBI was used to develop the scope and eligibility criteria of the review to ensure a clear and effective search strategy [42]. **Table 1** presents the inclusion and exclusion criteria.

**Table 1:** Inclusion and exclusion criteria for the scoping review.

	Inclusion criteria	Exclusion Criteria
**Participants**	Any population using GenAI-based chatbots for mental health problems, including prevention, support, or treatment.	No population or group will be excluded based on demographic or clinical characteristics, to capture a comprehensive overview of this emerging research area.
**Concept**	Papers describing the development, deployment, or evaluation of GenAI-based chatbot interventions (e.g., LLM-powered conversational agents) for mental health. This includes interventions that report on functionality, user experience (e.g., usability, satisfaction, trust), or safety (e.g., design safeguards, risk mitigation, adverse events).	Papers focused on non-generative AI Chatbots (e.g., rule-based or retrieval-based systems) or on other uses of Gen AI unrelated to chatbot interventions (e.g., diagnostic tools, or clinical decision support).
**Context**	Any setting where GenAI-based chatbots are being developed, tested, or deployed for mental health interventions. Includes healthcare, community, academic, self-help, or digital-only environments.	Given the novelty of the research area, studies/interventions will not be limited to any specific context.
**Study Design**	Primary quantitative, qualitative, and mixed methods study designs will be considered for inclusion, as well as descriptive studies that focus on chatbot design and/or deployment.	Scoping and systematic reviews, other review types and meta-analyses will be excluded.
**Sources**	Both peer-reviewed and grey literature (e.g., dissertations, conference proceedings) will be included. Only articles published in English language will be included.	Editorials, commentaries, opinion pieces textbook chapters, and non-scholarly media articles will be excluded. Non-English language papers will be excluded due to limited resources for translation.

### Search strategy

A preliminary search of MEDLINE was conducted to identify articles on the topic. Keywords and index (MeSH) terms identified from relevant articles were used to develop the full search strategy for MEDLINE (OVID) (S1 Appendix). This search strategy will be adapted to other selected databases: Scopus, PsycINFO, ACM Digital Library and IEEE Xplore. The databases were chosen to capture a broad range of sources from different disciplines related to the review objectives. PubMed was selected for its extensive coverage of publications in medicine and health sciences, while Scopus was chosen to include studies in relevant multidisciplinary areas such as science and technology, medicine, and social sciences. PsycINFO specializes in psychiatry and psychology related articles, making it essential for this review. The ACM Digital Library and IEEE Xplore were included because they index publications relating to AI and NLP application in mental health. The database search will be complemented by research-based search engines (Google Scholar and Consensus) to capture other relevant grey literature. The reference lists of all included sources of evidence will also be screened for additional studies.

### Selection of evidence sources

Following the search, all identified citations will be imported into Covidence with duplicates automatically removed [44]. Following a pilot test, titles and abstracts will be screened by two independent reviewers against the eligibility criteria. Potentially relevant sources will be retrieved in full, and the full text of selected citations assessed in detail against the eligibility criteria by the same independent reviewers. At this stage, reasons for exclusion of sources of evidence will be recorded and reported in the scoping review. Any disagreements that arise between the reviewers at each stage of the selection process will be resolved through discussion, or with an additional reviewer. The results of the search and the study inclusion process will be reported in full in the final scoping review and presented in a PRISMA-ScR flow diagram [42].

### Data extraction

Data will be extracted by two independent reviewers using a data extraction tool developed by the reviewers. The data extracted will include specific details about the participants, concept, context, study methods and key findings relevant to the review questions. **Table 2** lists important data that will be extracted from included studies. These will be used to develop a draft extraction form in *Covidence* which will be modified and revised as necessary during the data extraction process. As recommended by JBI, any disagreements that arise between the reviewers will be resolved through discussion, or with an additional reviewer, to achieve consensus [42]. Where appropriate, authors of papers will be contacted to request missing or additional data, where required.

**Table 2:** Proposed Data Extraction.

Study Characteristics	Intervention Details	GenAI-based Conversational Agent	User Experience measures	Safety Mechanisms
Author, Year, Country Type of evidence source, e.g., peer reviewed article, grey literature Article focus (e.g., development, evaluation, implementation) Study aim Study design Population Sample size Target condition(s) Primary outcomes	Setting Components Description Therapeutic approach Duration and Frequency	CA name Deployment platform, e.g., app, website Embodiment Type Underlying AI techniques, e.g., LLM model used Training data source/description Interaction mode (text, voice, multimodal)	Acceptability Usability Engagement/utilization Personalization/tailoring Therapeutic/working alliance	AI-based mechanisms, e.g., inclusive training datasets, finetuning, Retrieval Augmented Generation (RAG) Reinforcement Learning from Human Feedback (RLHF). Other pre-deployment safety measures, e.g., response accuracy testing, bias detection, data privacy and confidentiality Deployment phase/intervention delivery risk mitigation strategies, e.g., Human oversight, user education, crisis identification and management protocols.

### Data analysis and presentation

Data will be analysed and presented using descriptive statistics and narrative synthesis, focusing on the review objectives. Data visualization including summary tables, graphs and figures will be used to concisely present findings. Data extraction will be conducted within Covidence, which will also facilitate version control and audit trails. Extracted datasets will be exported to R (v4.2.3) for descriptive analysis and NVivo (v14) for qualitative synthesis, ensuring transparent data management and reproducibility [44,45]. Quantitative data will be summarized using descriptive statistics, including means, standard deviations, and frequency distributions, where applicable. No inferential or meta-analytic procedures will be performed, consistent with the scoping-review design. Qualitative data (e.g., user feedback and narrative findings) will undergo inductive thematic analysis following Braun and Clarke’s six-phase framework [46]. Coding will be performed independently by at least two reviewers, who will iteratively compare and refine themes through reflexive discussion until consensus is reached. An audit trail of coding decisions will be maintained to enhance transparency and trustworthiness [42].

Study characteristics (e.g., author, country, design, population, and context) will be summarized in structured tables and figures accompanied by a narrative overview. To map the GenAI-based chatbot interventions, a dedicated table will outline identified chatbots, their key features (e.g., deployment platform, interaction mode), and mental health problems they target.

User experience measures will be categorized and presented according to common themes such as acceptability, usability, engagement, and personalization, while safety mechanisms will be grouped into pre-deployment and delivery-focused strategies. To enhance conceptual clarity, pre-deployment (development-phase) safety mechanisms will be analyzed separately from deployment-phase risk mitigation strategies. The former includes model-training safeguards, bias and accuracy testing, and data-protection measures applied before user interaction. The latter captures real-world implementation safeguards such as human-in-the-loop oversight, user-support features, crisis-response protocols, and reporting of adverse events. This distinction will guide both data extraction and thematic synthesis. Overall, narrative synthesis will integrate findings across themes, highlighting innovative practices, safety considerations, and implications for future research, policy, and practice. The findings will inform the ethical design, evaluation, and regulation of GenAI tools for mental health care, offering timely guidance for developers, researchers, and policymakers seeking to ensure human-centered and safe deployment.

### Ethical considerations

This review involves analysis of publicly available literature and does not require ethical approval. Nonetheless, the broader ethical dimensions of GenAI mental health tools are recognized. Particular attention will be paid to how included studies address informed consent, data privacy, transparency, and the mitigation of potential psychological harm arising from chatbot use. These aspects will be highlighted in the synthesis to inform ethical best practices for future AI-enabled mental health interventions.

### Limitations

We acknowledge few anticipated limitations in this scoping review. First, the exclusion of non-English language publications may introduce language bias and limit the global comprehensiveness of the findings. Second, the expected variability across included studies in terms of study designs, reporting quality, and the varied operationalizations of key constructs such as user experience and safety may constrain the ability to directly compare results or synthesize findings quantitatively. Finally, given the rapidly evolving field of generative AI, relevant interventions may exist outside the academic literature, such as unpublished, proprietary, or inadequately described tools. This may affect the completeness of our review and highlights the need for ongoing updates as new evidence emerges. Despite these limitations, the review’s findings are expected to support the development of evidence-informed frameworks for responsible and equitable integration of generative AI in mental health interventions.

## Supporting information

S1 Appendix Initial Search for OVID MEDLINE (3/07/2024). (DOCX)

S2 Appendix PRISMA-P (Preferred Reporting Items for Systematic review and Meta-Analysis Protocols) 2015 checklist*. (PDF)
