# Supplementary material for: User experience and safety of generative AI-based mental health chatbots: Scoping review protocol
Source: PLoS One. 2026 Jan 23;21(1):e0341631. doi: 10.1371/journal.pone.0341631 (PMC12829926; doi:10.1371/journal.pone.0341631)
Supplement: S1 Appendix — (DOCX) [file pone.0341631.s001.docx]

## Supporting Information

### **S1** Appendix I

### Initial Search for OVID MEDLINE (3/07/2024)

|  | **Search Terms** | **Hits** |
| --- | --- | --- |
| 1 | exp artificial intelligence/ or exp natural language processing/ or exp neural networks, computer/ | 200886 |
| 2 | ("Artificial Intelligence" or "Generative Artificial Intelligence" or  "Generative AI*" or "Conversational Artificial Intelligence" or "Conversational AI*" or "Conversational Agent*" or "Conversational bot*" or "Conversational system*" or "AI Chatbot*" or "AI-Chatbot*" or "Chatbot*" or "Chat bot*" or "Chat-bot*" or "Relational agent*" or "Virtual agent*" or "Virtual coach*" or "Virtual Assistant*" or "Digital assistant*" or "Virtual therap*" or "virtual human*" or "AI-based*" or "AI based*" or "AI driven*" or "AI-driven" or "AI-enhanced*" or "AI enhanced*" or "AI-enabled" or "AI enabled" or "AI-powered" or "AI powered" or "Natural Language Processing" or "NLP or Large Language Model* or LLM* or Generative Pre?trained Transformer* or GPT* or ChatGPT* or Chat GPT*").mp. [mp=title, book title, abstract, original title, name of substance word, subject heading word, floating sub-heading word, keyword heading word, organism supplementary concept word, protocol supplementary concept word, rare disease supplementary concept word, unique identifier, synonyms, population supplementary concept word, anatomy supplementary concept word] | 91237 |
| 3 | 1 or 2 | 234666 |
| 4 | exp psychotherapy/ or exp behavior therapy/ or exp psychosocial intervention/ | 224655 |
| 5 | ("psychological intervention*" or "psychological therap*" or "psychological treatment*" or "psychosocial intervention*" or "psychosocial therap*" or "Behavio?ral therap*" or "cognitive behavio?ral therap*" or "CBT" or "iCBT" or "CBT-based" or "mental health intervention*" or "mental support" or "emotional support" or "Digital mental health" or "digital mental health intervention*" or "DMHI" or "e-mental health*" or "mental health app*" or "mental wellness app*" or "mental wellbeing app*" or "mental health platform*" or "online mental health*" or "telepsychiatry" or "digital psychotherap*" or "digital psychiatry").mp. [mp=title, book title, abstract, original title, name of substance word, subject heading word, floating sub-heading word, keyword heading word, organism supplementary concept word, protocol supplementary concept word, rare disease supplementary concept word, | 83114 |
|  | unique identifier, synonyms, population supplementary concept word, anatomy supplementary concept word] |  |
| 6 | 4 or 5 | 267433 |
| 7 | exp Mental Disorders/ | 1496908 |
| 8 | exp Substance-Related Disorders/ | 319909 |
| 9 | (anxiety or "anxiety disorder*" or depress* or bipolar* or "mood disorder" or phobia* or psychosis or psychotic* or "psychological stress" or "psychological distress" or "posttraumatic stress disorder*" or PTSD or "self-harm" or "self harm" or suicid* or "substance use*" or "substance abuse" or "drug abuse" or "alcohol abuse" or "alcohol misuse" or "mental disorder*" or "mental illness*").mp. [mp=title, book title, abstract, original title, name of substance word, subject heading word, floating sub-heading word, keyword heading word, organism supplementary concept word, protocol supplementary concept word, rare disease supplementary concept word, unique identifier, synonyms, population supplementary concept word, anatomy supplementary concept word] | 1383661 |
| 10 | 7 or 8 or 9 | 2267557 |
| 11 | 3 and 6 and 10 | 521 |
